# Effect of Compensatory Mechanisms on Postural Disturbances and Musculoskeletal Pain in Elite Sitting Volleyball Players: Preparation of a Compensatory Intervention

**DOI:** 10.3390/ijerph181910105

**Published:** 2021-09-26

**Authors:** Eliza Gaweł, Anna Zwierzchowska

**Affiliations:** Institute of Sport Sciences, The Jerzy Kukuczka Academy of Physical Education in Katowice, 40-065 Katowice, Poland; a.zwierzchowska@awf.katowice.pl

**Keywords:** spinal curvature, Paralympic volleyball, compensation strategy, thoracic hyperkyphosis, adapted training, low back pain

## Abstract

The aim of the study was to identify the effect of compensatory mechanisms on the prevalence of sagittal spinal curvature deformity and musculoskeletal pain and to assess the interrelationships between those components in sitting volleyball players. Twenty-one elite Polish sitting volleyball players (age = 34.1 ± 7.5, BM = 77.9 ± 16.0) participated in the study in which direct participatory systematic observation and a non-invasive method were used. Both objective (anthropometric, spinal curvature–Idiag M360) and subjective (musculoskeletal ailments–NMQ = 7) measurements were performed. The Statistica 13.3 software package was used for statistical analyses. The neck, lower back (43%), and upper back (38%) were the most frequently reported painful areas. Of all participants, 76% reported sagittal spinal deformities. In the habitual position, the results indicated moderate correlations (r = 0.5, *p* < 0.05) between the lumbar concavity of the back and low back pain (LBP) and between thoracic convexity and LBP (r = 0.4, *p* < 0.05). Internal and external compensation have an effect on the prevalence of spinal curvature deformities in the sagittal plane, with thoracic hyperkyphosis (38%) and lumbar hyperlordosis (33%) being the most common. More severe lower and upper back pain were correlated with greater angles of thoracic kyphosis and lumbar lordosis in the habitual position.

## 1. Introduction

Body posture is affected by many factors. However, it is mostly determined by the shape of the spine [1], which comprises the opposing curves, i.e., kyphosis and lordosis. In a balanced spine, thoracic kyphosis and lumbar lordosis are intrinsically related, and therefore, one curvature responds to the development or disturbance of the other. Furthermore, the pelvic position strongly interacts with the spinal shape by controlling the sagittal balance between the aforementioned curvatures [2,3].

Since physical activity has been acknowledged to impact spinal curvature, athlete body posture has become an area of interest for numerous scientists [1,4,5]. According to Grabara [4], sport-specific training causes multiple changes in an athlete’s body build and posture, which leads to the use of adaptative strategies, even if they are not necessarily beneficial. According to Paralympic athletes, two interdependent (internal and external) mechanisms are important. Internal compensation is a necessary yet only partly beneficial compensation strategy due to a congenital or acquired impairment. However, it mostly disturbs the proper function of movement in the human body, such as trunk rotation or pelvic flattening. On the other hand, external compensatory mechanisms are developed due to the specificity of the sport practiced, which is known as the body’s adaptation to the sport-specific movements. Despite the fact that the abovementioned compensatory mechanisms are essential for Paralympic athletes to keep the upright position and sagittal balance of the spine (internal strategy) and to meet the requirements of the sport-specific technique (external strategy), there are several disadvantages that need to be addressed. With the focus on sport-specific functional and structural movements and high training loads, athletes develop muscular dystonia and structural changes in the skeletal system; as a consequence, athletes are prone to musculoskeletal ailments [6].

As pain is known to be one of the most common problems in professional sport [7,8], there is a need for studies that address the possibilities to avoid or reduce musculoskeletal pain and the negative effects of body compensation strategies, especially in Paralympic sport. These problems are especially important in sitting volleyball players since the vast majority of them have lower body impairments [9], especially amputations or limb deficiencies. These types of disabilities activate several internal compensatory mechanisms because of the changed position of the center of gravity of the body [10]. Furthermore, in sitting volleyball players, the upper limbs are constantly overloaded because of sport-specific movements, e.g., services or attacks [11], and the necessity of playing in a sitting position. Therefore, external compensatory mechanisms such as muscle imbalance are often observed in this group [11].

It should be noted that a disabled athlete cannot control his or her physiological limitations caused by a congenital or acquired disability. However, the athlete can choose to avoid or manage musculoskeletal pain and attempt to minimize the negative effects of internal and external adaptative strategies.

To the best of our knowledge, no studies in the currently available scientific literature have examined musculoskeletal pain in relation to spinal curvatures. Therefore, the aim of our study was to evaluate the effect of internal and external compensatory mechanisms on the prevalence of spinal curvature deformities in the sagittal plane and musculoskeletal pain and to assess the interrelationships between the aforementioned components. We hypothesized that lower limb disability and sitting volleyball training impact the spinal curvature in the sagittal plane. Furthermore, it was established that spinal deformities are interrelated with the prevalence of musculoskeletal pain. We assumed that the findings of our study would indicate the need for developing an adapted training program with compensatory proprioceptive exercises that could be implemented in the future as an intervention for sitting volleyball players.

## 2. Materials and Methods

### 2.1. Participants

The study examined twenty-one elite Polish sitting volleyball players (*n* = 6 women; *n* = 15 men) from the Polish national team. The inclusion criteria were (a) at least a minimal disability (MD) according to the World ParaVolley classification and (b) no neuromuscular or musculoskeletal disorders. Table 1 shows a description of the participants.

The amputee group used prostheses (*n* = 11) or orthopedic crutches (*n* = 2) in the activities of daily living and locomotion. Only one athlete had a bilateral amputation above the knees and used a wheelchair in everyday life. The athletes from the Les Autres group used prostheses (*n* = 3), orthopedic crutches (*n* = 1), or no supportive equipment (*n* = 3).

The measurements were carried out during a five-day national team training camp in the Jerzy Kukuczka Academy of Physical Education in Katowice, Poland. The participants were informed about the advantages and disadvantages of the study and provided written informed consent. The research protocol was approved by the Bioethics Committee for Scientific Research at the Academy of Physical Education in Katowice, Poland (No. 9/2012) and met the ethical standards of the Declaration of Helsinki, 2013. The participants were allowed to withdraw from the study at any moment. Furthermore, they were instructed to keep their normal dietary and sleeping habits for 24 h before the study.

### 2.2. Methods and Measurements

**Figure 1:**
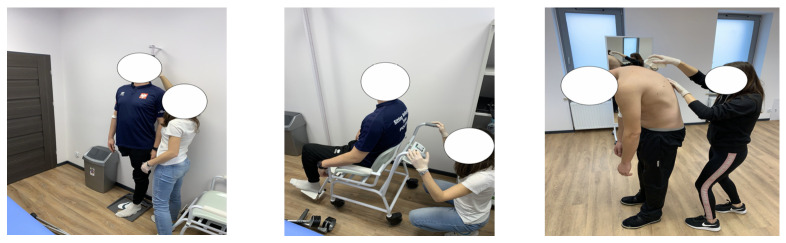
Examples of anthropometric measurements and spinal curvature measurements.

A direct participatory systematic observation method was used in the study, which requires the direct participation of the studied group and the researcher, who directly assesses the participants. The Nordic Musculoskeletal Questionnaire [12] was employed to assess the prevalence and locations of musculoskeletal pain from the last seven days (NMQ = 7) and included the following nine body parts: neck, shoulders, upper back, elbows, wrists, low back, hips/thighs, knees, and ankles/feet. Before completing the questionnaire, the athletes were instructed not to report phantom pain. Next, anthropometric measurements were taken (Figure 1). A wall-mounted stadiometer with a centimeter scale was used for body height (BH) measurements, including the wheelchair user who was able to stand on amputation stumps. Body mass (BM) was evaluated with a chair weight. Hip (HC) and waist (WC) circumferences were measured with the use of anthropometric tape on bare skin, in a lying position and according to the recommended anthropometric techniques, i.e., HC, around the greatest convexity of the gluteal muscles below the iliac ala and WC, at the midpoint between the superior iliac crest and the lowest rib [13]. Spinal curvatures were evaluated using a non-invasive method with a Medi Mouse (Idiag M360) (Figure 1), which ensures producibility, even if two different researchers perform the measurement. The examinations were conducted in three different trunk positions, i.e., sagittal standing (arms in the habitual position), sagittal standing flexion (arms in free stance), and extension (arms crossed at the shoulders, elbows up). Before the measurements, all procedures were demonstrated and explained. The measurements started by putting the Medi Mouse at the C_7_ level. Next, the device was moved with constant speed up to the S_5_ level. All measurements were automatically recorded on a computer with Idiag M360 software, which indicates the values from anteroposterior spinal curvatures, physiological values, the differences between them, and the type of sagittal spinal deviation (thoracic hypo/hyperkyphosis, lumbar hypo/hyperlordosis) based on individual BH, BM, gender, age, and the values from anteroposterior spinal curvatures in a habitual position.

### 2.3. Statistical Analysis

Statistical analyses were performed with Statistica 13.3 software package (TIBCO Software Inc., Tulsa, OK, USA). Results are presented as means ± SD for normally distributed data and as geometric means with a 95% confidence interval. The prevalence of faulty body posture in the sagittal plane and its relation to symptoms in different parts of the musculoskeletal system in the group of Paralympic athletes was compared using statistic structure index (SSI).

Pearson’s correlation coefficients were computed for the characteristics of NMQ = 7 and the parameters from Medi Mouse, recorded in different positions (sagittal standing upright, sagittal standing flexion, and extension) for the group of Paralympic athletes. The normality of thoracic kyphosis and lumbar lordosis distributions was verified with the Chi-square test. The level of statistical significance was set at 5%.

## 3. Results

Table 2 presents objective results obtained from the Medi Mouse (thoracic kyphosis and lumbar lordosis angles, physiological values, and differences between actual and physiological values of the aforementioned curvatures in three positions in the sagittal plane) and subjective results of the prevalence and location of musculoskeletal pain based on the NMQ = 7. Table 3 shows the results of statistical correlations between NMQ = 7 and angles of anteroposterior spinal curvatures and differences between physiological norms of thoracic kyphosis and lumbar lordosis (sagittal standing, sagittal standing flexion, sagittal standing extension) based on the Medi Mouse.

The neck (43%), lower back (43%), and upper back (38%) were the most often reported painful areas, whereas the lowest prevalence of pain was found for shoulders, elbows, and ankles/feet (19%). Furthermore, based on the individual reports obtained from the Idiag M360 software, sagittal spinal deviations were found in the vast majority of sitting volleyball players (76%), i.e., thoracic hyperkyphosis (38%), lumbar hypolordosis (33%), thoracic hypokyphosis (19%), and lumbar hyperlordosis (14%).

In the habitual position, the results indicate moderate correlations (r =0.5, *p* < 0.05) between the deepening of lumbar lordosis and low back pain (LBP) and between deepening thoracic kyphosis and LBP (r = 0.4, *p* < 0.05). Similar moderate relationships (r = 0.4, *p* < 0.05) were found for the sagittal standing extension. Moreover, a correlation between neck pain and the thoracic kyphosis angle was found in both sagittal standing flexion and extension (r = 0.4, *p* < 0.05). Furthermore, the statistical analysis showed a moderate relationship between the prevalence of upper back pain and physiological norms of thoracic kyphosis (r = 0.4, *p* < 0.05).

## 4. Discussion

This study aimed to evaluate the effect of internal and external compensation on the prevalence of musculoskeletal pain and postural defects in elite Polish sitting volleyball players and to assess the interrelation between the aforementioned components. A major finding of this study was that the deeper the thoracic kyphosis and lumbar lordosis angles were, the higher was the prevalence of LBP reported in sagittal standing and sagittal standing extension. Furthermore, neck pain occurred more frequently in athletes with a deeper angle of thoracic kyphosis in both sagittal standing flexion and extension. Moreover, the statistical analysis showed direct proportional associations between upper back pain and physiological norms of the thoracic kyphosis angle.

The results of this study fully support our initial hypothesis and confirm that both lower limb deficiency or disability and sitting volleyball training impact the prevalence of spinal deviations in the sagittal plane. Furthermore, our results point out that anteroposterior spinal curvature deviations are interrelated with musculoskeletal pain, especially in the lower back (43%), neck (43%), and upper back (38%).

Many studies have been carried out to assess the prevalence of musculoskeletal pain in elite able-bodied volleyball players, in whom the lower back was found to be the most common location of pain [14,15,16,17,18]. Moreover, studies by Movahed et al. [19] showed that a greater angle of lumbar lordosis in a habitual position is associated with a higher prevalence of LBP in volleyball athletes. This result corresponds with our findings; however, LBP also contributed to a deepening of lumbar lordosis in sagittal extension and a deepening of thoracic kyphosis in both sagittal standing and extension. These findings may be related to both internal and external compensatory mechanisms that might have impacted muscle imbalance and caused spinal deviations in the sagittal plane, observed in 76% of Paralympic athletes.

It needs to be noted that sagittal balance depends on the angles of thoracic kyphosis and lumbar lordosis, whereas pelvic position strongly interacts with spinal shape by regulating the sagittal balance between the curves [2]. Moreover, the available scientific studies indicate that lower limb/limbs amputation disturbs body biomechanics [10,20]; thus, to maintain balance and upright posture, the human system must activate internal compensatory mechanisms, even if this is not fully beneficial. Unilateral limb amputation/impairment affects the spinal curvature mostly by deepening thoracic kyphosis and flattering lumbar lordosis, as reported in the vast majority of sitting volleyball players.

Furthermore, adaptation to sitting volleyball training, i.e., external compensation strategy, should also be mentioned. In the currently available scientific literature, several studies have analyzed the impact of sport-specific training on the prevalence of anteroposterior spinal curvature deviations in volleyball players [4,21,22]. However, it is difficult to find a study that confirms such effects in Paralympic athletes, especially amputees. Nevertheless, Grabara [4,21] indicated volleyball training as a factor activating the external compensation strategy by deepening thoracic kyphosis and consequently flattening or deepening lumbar lordosis, which is consistent with the results of our study and indicates lumbar hypolordosis (33%) and thoracic hyperkyphosis (38%) as the most common sagittal spinal deformities in sitting volleyball players. Additionally, upper back pain appeared mostly in Paralympic athletes with incorrect values of thoracic kyphosis (38%), which corresponds with the studies of Fett et al. [23] who found the relationships between volleyball training and the high prevalence of upper back pain.

Moreover, a specific sitting position that is taken while playing sitting volleyball should be emphasized. According to the World ParaVolley rules, players can move on the court by sliding or using their upper limbs; however, at least one part of the player’s buttocks must remain on the floor while the ball is in play [24]. Because of the forced sitting position, players have a tendency to overload upper limbs and develop muscular imbalances [11] that might strongly contribute to both a deepening of thoracic kyphosis and pain in an athlete’s upper body, which was found in this study.

To date, plenty of research has analyzed the prevalence of musculoskeletal pain in volleyball players, which was found mostly in the upper and lower back [25,26,27]. However, few studies have indicated neck pain as a significant problem in volleyball players [18,24]. Nevertheless, it is hard to find a study that demonstrates the relationships between neck pain and sagittal spinal deformities. Our results have shown a moderate correlation between neck pain and thoracic hyperkyphosis in the sagittal standing flexion and extension, which might be a consequence of the aforementioned compensation strategies.

The findings of our study may be taken into consideration by sitting volleyball players, who are characterized by the high prevalence of musculoskeletal pain and spinal curvature deformities. Therefore, we recommend, especially for Paralympic athletes, an adapted compensatory training program with proprioceptive exercises (Table A1, Appendix A), which was programmed based on the obtained subjective and objective results to prevent or reduce deformities of spinal curvature and musculoskeletal pain.

### Limitations

It should be noted that our study has several limitations that need to be acknowledged. Firstly, even though we explored the entire men’s (n = 15) and women’s (n = 6) Polish sitting volleyball national team, the group of participants consisted in large part of men, which leads to incomplete inference, especially regarding differences in the prevalence of spinal curvature deformities between the two genders. However, it should be noted that the female sitting volleyball national team made its debut twelve years after the male team [28], which may be associated with a smaller number of elite female athletes. Furthermore, we examined athletes only from two disability groups (amputees, Les Autres).

Secondly, the programmed compensatory exercise intervention has not yet been verified. However, it was developed according to the newest trends in kinesitherapy and corrective methodology. Simultaneously, the authors are planning its verification after extending the group of participants to those with other disabilities, e.g., spinal cord injuries. Such studies will provide important information to improve athletic performance through the prevention of musculoskeletal pain and to reduce the negative effects of internal and external compensation strategies.

## 5. Conclusions

1. Internal and external compensation have an effect on the prevalence of deformities of spinal curvature in the sagittal plane, with thoracic hyperkyphosis (38%) and lumbar hyperlordosis (33%) being the most common.
2. The neck, lower back (43%), and upper back (38%) were the most frequent painful areas in sitting volleyball players. More severe LBP and upper back pain were correlated with a greater angle of thoracic kyphosis and lumbar lordosis in the habitual position.
3. The findings of the study have inspired the programming of an adapted compensatory training program to decrease and prevent the abovementioned spinal deformities and musculoskeletal pain.

## Figures and Tables

**Table 1:** Characterization of the sitting volleyball players.

Characteristics (*n* = 21; nW = 6, nM = 15)	Mean ± SD or Percentage
Age (years)	34.1 ± 7.5
Body mass (kg)	77.9 ± 16.0
Body height * (cm)	178.6 ± 0.1
Hip circumference (cm)	103.3 ± 10.0
Waist circumference (cm)	89.3 ± 11.1
BMI with a limb deficiency (*n* = 16)	23.7 ± 4.9
BMI without a limb deficiency (*n* = 5)	24.9 ± 1.9
BAI* (%)	24.8 ± 3.8
Disability time (years)	20.2 ± 11.1
Experience in sitting volleyball training (years)	8.1 ± 7.6
**Medical Classification**	
Amputees in general	62%
Amputees–A1	5%
Amputees–A2	28.5%
Amputees–A4	28.5%
Les Autres in general	38%
Les Autres–LA5	33%
Les Autres–LA6	5%

*n*—total number of participants; nW—number of women; nM—number of men; SD—standard deviation; * excluded bilateral amputation; BMI—body mass index; BAI—body adiposity index; A1—bilateral thigh amputation; A2—lateral thigh amputation; A4—lateral shank amputation; LA5—limited efficiency in one lower limb; LA6—incapacity in one upper limb.

**Table 2:** Means and standard deviations (SD) of angles of anteroposterior spinal curvatures (°) in three sagittal positions and the prevalence (%) and locations of musculoskeletal pain based on NMQ = 7.

Spinal Curvature Measurements: Sagittal Plane (n = 21; nW = 6, nM = 15)	Mean ± SD (°)	Body Parts (NMQ = 7)	(*n* = %)
TK–sagittal standing	37.1 ± 18.8	Neck	43%
Physiological values	38.8 ± 18.9		
Difference	1.7 ± 2.6		
TK–sagittal standing flexion	49.6 ± 23.7	Shoulders	19%
Physiological values	51.2 ± 24.9		
Difference	3.7 ± 5.0		
TK–sagittal standing extension	30.4 ± 15.3	Upper back	38%
Physiological values	30.2 ± 16.2		
Difference	2.5 ± 3.3		
LL–sagittal standing	18.9 ± 13.5	Elbows	19%
Physiological values	20.5 ± 14.2		
Difference	1.6 ± 2.7		
LL–sagittal standing flexion	21.8 ± 13.5	Wrists	24%
Physiological values	20.0 ± 12.2		
Difference	3.7 ± 5.0		
LL–sagittal standing extension	29.8 ± 16.4	Lower back	43%
Physiological values	29.6 ± 15.5		
Difference	2.6 ± 3.3		
		Hips/ties	24%
		Knees *	29%
		Ankles/feet *	19%

TH–thoracic kyphosis; LL–lumbar lordosis; n–total number of participants; nW = number of women; nM = number of men; SD–standard deviation; NMQ = 7–Nordic Musculoskeletal Questionnaire from last seven days; *—one participant did not respond due to bilateral amputation above the knees.

**Table 3:** The results of statistical correlations between the prevalence and location of musculoskeletal pain (NMQ = 7) and angles of anteroposterior spinal curvatures and differences between physiological norms of thoracic kyphosis and lumbar lordosis angles (Medi Mouse).

Body Parts (NMQ = 7)	Sagittal Standing				Sagittal Standing Flexion				Sagittal Standing Extension			
	TH	±	LL	±	TH	±	LL	±	TH	±	LL	±
Neck	0.3	0.3	SI	SI	0.4	SI	−0.09	SI	0.4	0.2	0.2	SI
Arms	0.3	SI	SI	SI	0.3	SI	SI	SI	SI	SI	SI	SI
Upper back	SI	0.4	0.3	0.3	0.16	SI	0.15	−0.1	0.15	SI	0.2	SI
Low back	0.4	−0.2	0.5	−0.2	0.2	SI	SI	−0.1	0.4	−0.2	0.4	−0.2

TH—thoracic kyphosis; **±**—the difference between physiological norm and TH or LL angle; SI—statistically insignificant; LL—lumbar lordosis.

## Data Availability

The data presented in the study are available on request from the corresponding author.

## Appendix A

**Table A1:** Adapted training program with compensatory, proprioceptive exercises for sitting volleyball players.

Exercise Number	The Kind of Exercise	Compensatory Influence	Initial Number of Series and Repetitions	Exercise Process–Version A (Easy)	Exercise Process–Version B (Difficult)	Comments
1.	Mobilization & breathing exercise (thoracic segment)	- Strengthening breathing muscles (inspiratory/expiratory) - Stretching chest muscles - Thoracic spine mobilization	2 × 10	I.P. 90/90 sit, arms behind the neck, elbows inside. Movement: 1–4. Deep breath through the nose with progressive backward trunk bending and side elbow abduction. 5–8. Frontal trunk bending with deep exhale through the mouth and inside elbow adduction. E.P. = I.P.	I.P. 90/90 sit, arms crossed on the chest Movement: 1–4. Deep breath through the nose while going up with progressive side arm abduction and backward trunk bending. 5–8. Frontal trunk bending with a deep exhale through the mouth and crossing the arms across the chest while going down to the initial position. E.P. = I.P.	- The inspiratory and expiratory phase should last 4 seconds.
2.	Mobilization & breathing exercise (upper limbs)	- Enhancement of the range of motion in the humeral joint - Stretching chest muscles - Strengthening breathing muscles (inspiratory/expiratory)	The side without a disfunction 2 × 10 The side with a disfunction 2 × 15	I.P. Lying sideways (left side), legs bent at the knee joints, arms in the front, hands together. Movement: 1–4. Side move of the right arm from the front to the right side while turning the head to the right and taking a deep breath through the nose. 5–8. Side move of the right arm from the right side to the front while turning the head to the left and exhaling deeply through the mouth. E.P. = I.P.	I.P. Lying sideways (left side), legs bent at the knee joints, arms in the front, hands together. Movement: 1–4. Side move of the right arm from the front to the back with a hand rotation to the dorsal position while turning the head to the right and taking a deep breath through the nose. 5–8. Side move of the right arm from the back to the front with a hand rotation to the areal position while turning the head to the left and exhaling deeply through the mouth. E.P. = I.P.	-Versions A and B are performed on both sides. - The inspiratory and expiratory phase should last 4 seconds.
3.	Mobilization exercise (lower limbs)	- Enhancement of the range of motion in the hip joints - Stretching the iliolumbar and quadriceps muscles	The side without a disfunction 2 × 10 The side with a disfunction 2 × 15	I.P. Seated frontal bend, arms on the floor. Movement: 1. Right leg abduction to the floor. 2. Right leg adduction to the initial position. 3. Left leg abduction to the floor. 4. Left leg adduction to the initial position. E.P. = I.P.	I.P. Seated frontal bend, arms to the side. Movement: 1. Right and left leg abduction to the floor (movement to the right). 2. Leg adduction to the initial position. 3. Left and right leg abduction to the floor. (movement to the left) 4. Leg adduction to the initial position. E.P. = I.P.	
4.	Activation exercise (upper limbs)	- Rotator cuff activation - Balance the shoulder blade rhythm - Thoracic spine activation	Version A 3 × 10 Version B 2 × 8	I.P. Lying on the front, arms overhead, hands vertical, forehead on the floor. Movement: 1. Raising the arms upwards. 2. Lowering the arms downwards while bending the elbow joints and rotating the hands to the dorsal position. 3. Raising the arms upwards while extending the elbow joints and rotating the hands to the initial position. 4. Lowering arms downwards. E.P. = I.P.	I.P. Lying on the side, arms overhead, hands vertical, forehead on the floor. Movement: 1. Raising the arms upwards. 2. Adduction of the arms sideways. 3. Internal hand rotation to the dorsal position 4. Bending arms at the elbow joints with a side move to the thoracic spine. 5. Lowering the elbows downwards. 6. Raising the elbows upwards with an arm extension to the side (hands in internal rotation). 7. Lifting the arms upwards from the front while rotating the hands to the initial position 8. Lowering arms downwards. E.P. = I.P.	- Before each repetition–retraction and depression of the scapula.
5.	Activation exercise (lower limbs)	- Gluteus muscle activation - Central stability	The side without a disfunction 3 × 6 The side with a disfunction 3 × 10	I.P. 90/90 sit, front foot in the dorsal position, arms between the knee joint. Movement: 1. Raising the front leg (bent at the knee joint) upwards. 2. Lowering the front leg to the initial position. E.P. = I.P.	I.P. Four-point kneeling Movement: 1. Moving the left leg backward. 2. Holding. 3. Moving the left leg sideways. 4. Lowering the left leg downward. 5. Moving the left leg sideways. 6. Moving the left leg backward. 7. Holding. 8. Lowering the left leg to the initial position. E.P. = I.P.	- Before each repetition–scapula protraction. - Versions A and B are performed on both sides. -Version B–lumbar spine and pelvis without extreme rotation.
6.	Activation exercise (trunk)	- Abdominal muscles activation	Version A 2 × 8 Version B The side without a disfunction 2 × 8 The side with a disfunction 2 × 12	I.P. Lying on the front, arms crossed on the chest. Movement: 1. Raising the trunk upwards. 2–3. Holding. 4. Lowering the trunk downwards. 5. Raising and turning the trunk to the left side. 6. Lowering the trunk to the initial position. 7. Raising and turning the trunk to the right side. 8. Lowering the trunk to the initial position. E.P. = I.P.	I.P. Lying on the back with the legs bent at the knee joints (feet in a dorsal position), left arm upwards, right arm on the left knee joint– pushing slightly. Movement: 1. Lowering the left arm and the right leg downwards (to the straight body level). 2. Raising the left arm and the right leg upwards to the initial position. E.P. = I.P.	- Verison A: raising and lowering the trunk, vertebra by vertebra. - Version B: the exercise is performed on both sides, and the lumbar spine should globally touch the floor during the entire motor activity.
7.	Directional exercise (thoracic segment)	- Stretching chest muscles - Strengthening the latissimus dorsi muscle and teres major muscle - Strengthening the rotator cuff - Balance the shoulder blade rhythm	3 × 10	I.P. Kneeling sit, arms downwards, hands are holding a resistance band (hips widthways). Movement: 1. Moving the right arm from the front to the back. 2. Moving the right arm from the back to the front. E.P. = I.P.	I.P. Kneeling sit, arms downwards, hands are holding a resistance band (hips widthways). Movement: 1. Moving the arms from the front to the back. 2. Moving the arms from the back to the front. E.P. = I.P.	- Before each repetition–retraction and depression of the scapula. - During the exercise, there should not be any compensation with trunk arcuation in the lumbar spine - Version A: the exercise is performed on both sides. During the entire movement activity, the band should be maintained in a slight tension. - Version B: during the entire movement activity, the band should be maintained with the same tension.
8.	Directional exercise (thoracic segment)	- Stretching chest muscles - Strengthening the quadratus lumborum muscle, rhomboid muscle, and latissimus dorsi muscle - Strengthening the rotator cuff - Balance the shoulder-blade rhythm	3 × 10	I.P. 90/90 sit, arms in the front (head level), hands are holding the resistance band (shoulders widthways). Movement: 1. Pulling the band from the front to the side. 2. Returning to the initial position. E.P. = I.P.	I.P. 90/90 sit, arms upwards, hands are holding the resistance band (shoulders widthways). Movement: 1. Pulling the band downwards. 2. Returning to the initial position. E.P. = I.P.	- Before each repetition–scapulas retraction and depression. - During the entire movement activity, the band should be kept with a slight tension.
9.	Directional exercise (lumbar segment)	- Strengthening gluteus muscles - Strengthening serratus anterior muscle, superior and external oblique muscles - Central stability	The side without a disfunction 3 × 10 The side with a disfunction 3 × 15	I.P. Lying sideways (left side) with bent legs, the left arm bent at the elbow joint and lying on the forearm, the right arm bent at the elbow joint (on the trunk level), Movement: 1. Raising trunk upwards while raising the right leg upwards. 2. Holding the trunk with a right arm abduction and while turning the trunk to the right. 3. Holding the trunk with a right arm adduction and a trunk flexion to the body level. 4. Lowering the trunk and legs to the initial position. E.P. = I.P.	I.P. Lying sideways (left side) with bent legs, the left arm bent at the elbow joint and lying on the forearm, the right arm upwards. Movement: 1. Raising the trunk and right leg upwards. 2. Holding the trunk while lowering the right leg. 3. Holding the trunk while raising the right leg upwards. 4. Lowering the trunk and legs to the initial position. E.P. = I.P.	- Before each repetition–retraction and depression of the scapula. - During the motor activity, the trunk should be stabilized.
10.	Directional exercise (lumbar segment)	- Strengthening the rectus abdominis muscle (version A) and superior and external oblique muscles (version B) - Stretching the quadratus lumborum muscle	3 × 30 s.	I.P. Lying on the back with raised legs bent at the knee joints, arms upwards. Movement: 1. Raising the trunk while moving the arms downwards (knee joints level, hands vertical). 2. Holding (30 s). 3. Lowering the legs to the initial position. 4. Moving the arms upwards. E.P. = I.P.	I.P. Lying on the back with raised legs bent at the knee joints, arms upwards. Movement: 1. Raising the trunk while moving the arms downwards (knee joints level, hands in areal position). *Alternative 2–3 (30 s.)* 2. Trunk flexion to the left side. 3. Trunk flexion to the left side. 4. Lowering the legs to the initial position while moving the arms upwards. E.P. = I.P.	- During the exercise, the lumbar spine should globally touch the floor. When a lumbar lordosis accentuation can be seen, the exercise should be stopped.

I.P.—internal position; E.P.—end position.
